# From a traditional medicine monomer to a modern neurotherapeutic scaffold: a review of SAR-Driven tetramethylpyrazine derivatives for cerebrovascular and cognitive health

**DOI:** 10.3389/fphar.2025.1653056

**Published:** 2025-08-21

**Authors:** Xiaodi Wang, Muhan Cao, Yi Xu, Xifei Yang, Qinghua Hou

**Affiliations:** ^1^ Department of Neurology, Clinical Neuroscience Center, The 7th Affiliated Hospital, Sun Yat-Sen University, Shenzhen, China; ^2^ Department of Rehabilitation Medicine, The 7th Affiliated Hospital, Sun Yat-Sen University, Shenzhen, China; ^3^ Shenzhen Key Laboratory of Modern Toxicology, Shenzhen Medical Key Discipline of Health Toxicology (2020-2024), Shenzhen Center for Disease Control and Prevention, Shenzhen, China

**Keywords:** tetramethylpyrazine, pharmacology, pharmacodynamics, structure-activity relationships, cerebrovascular function, cognition

## Abstract

Tetramethylpyrazine (TMP), a bioactive alkaloid isolated from the traditional Chinese medicine *Ligusticum wallichii* (*Chuanxiong)*, has gained significant attention for its therapeutic potential in cerebrovascular diseases and cognitive impairment, mainly due to its antioxidant, anti-inflammatory, and anti-apoptotic properties. However, its clinical application is often limited by suboptimal pharmacokinetic characteristics and modest potency. This review highlights recent advancements in the structure-activity relationship (SAR) optimization of TMP, focusing on its derivatives’ neuroprotective efficacy and vascular benefits. We specifically emphasize the clinical translational potential of several TMP derivatives, such as T-006, TMP-nitrone hybrids (e.g., TN-2), TMP-piperazine derivatives, and TMP-phenolic acid hybrids (e.g., T-VA). These compounds exhibit markedly improved drug-like properties, including enhanced lipid solubility, oral bioavailability, blood-brain barrier (BBB) permeability, and multi-target neuroprotective actions. Additionally, we critically examine the challenges these TMP derivatives face in clinical translation, such as metabolic instability, hepatotoxicity, and formulation challenges, while discussing current strategies to address these issues. The review concludes by emphasizing the significant promise of these next-generation TMP derivatives as therapeutic candidates for cerebrovascular and neurodegenerative disorders, and their need for further preclinical and clinical exploration to fully realize their therapeutic potential.

## 1 Introduction

**FIGURE 1:**
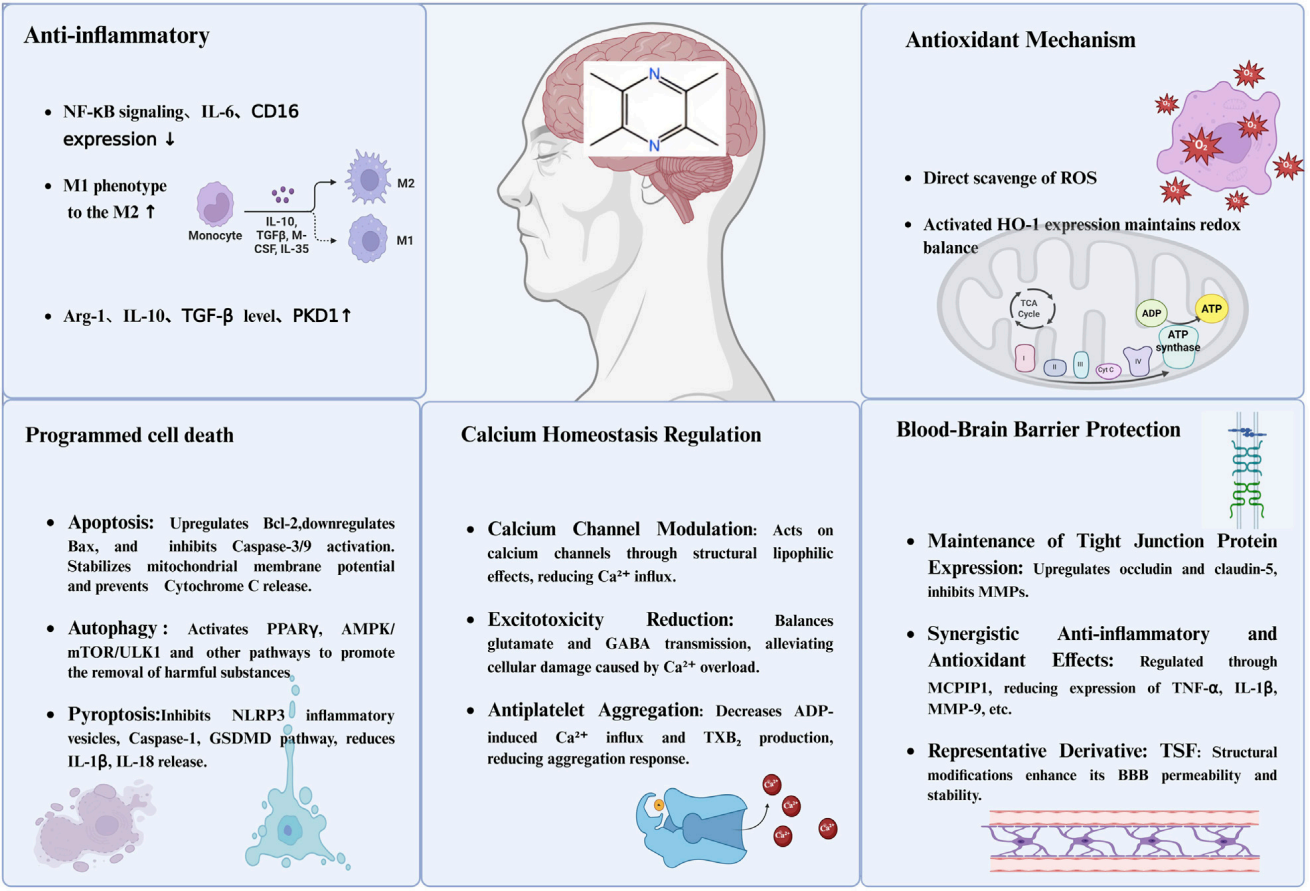
The primary neuroprotective mechanisms of TMP. Notes: This figure illustrates the multifaceted neuroprotective mechanisms of TMP in the central nervous system. (1) Anti-inflammatory effects: TMP downregulates NF-κB signaling, IL-6, and CD16 expression, promotes M1-to-M2 macrophage polarization, and increases levels of anti-inflammatory mediators such as IL-10, TGF-β, and PKD1. (2) Antioxidant mechanisms: It directly scavenges reactive oxygen species (ROS) and induces HO-1 expression to maintain redox homeostasis. (3) Programmed cell death inhibition: TMP modulates apoptosis by upregulating Bcl-2 and inhibiting caspase pathways, promotes autophagy via PPARγ and AMPK/mTOR/ULK1 signaling, and suppresses pyroptosis by inhibiting NLRP3 inflammasome and GSDMD activation. (4) Calcium homeostasis regulation: It modulates calcium channels to reduce Ca^2+^ influx, prevents excitotoxicity by balancing glutamate and GABA transmission, and reduces platelet aggregation by inhibiting TXB_2_ production. (5) BBB protection: TMP enhances tight junction protein expression (occludin and claudin-5), suppresses MMP activity, and exhibits synergistic anti-inflammatory and antioxidant effects, thereby improving BBB integrity and permeability.

Cerebrovascular and cognitive disorders constitute a mounting global health crisis, contributing substantially to disability, mortality, and socioeconomic burden worldwide. The search for effective neurovascular therapeutics remains a pressing priority, especially given the limitations of existing pharmacological options in mitigating neuronal loss and vascular dysfunction. Among natural products, tetramethylpyrazine (TMP)––the principal active alkaloid extracted from *Ligusticum chuanxiong Hort*––has garnered considerable attention for its multifaceted neurovascular protective properties (Yuan et al., 2020; Li L. et al., 2022) (Figure 1).

Extensive preclinical and clinical studies have substantiated TMP’s capacity to ameliorate ischemic stroke, cognitive impairment, and various neurodegenerative conditions through anti-inflammatory, antioxidant, and vasodilatory mechanisms (Lian et al., 2023; Zhao et al., 2016; Lin et al., 2022; Zhang et al., 2021). However, its therapeutic translation is critically hampered by pharmacokinetic drawbacks—most notably, a rapid systemic clearance and low oral bioavailability (Ge et al., 2020)—which limit sustained brain exposure and clinical efficacy.

In response, significant advances in medicinal chemistry have focused on the rational design and structural modification of TMP. Contemporary efforts have yielded diverse TMP derivatives—including piperazine conjugates, cinnamic acid hybrids, and amide analogues (Zou et al., 2018)—that not only augment pharmacokinetic performance but also expand the therapeutic landscape through novel biological activities and mechanisms of action. These derivatives show promise in modulating cerebrovascular integrity, maintaining BBB function, attenuating neuroinflammation, and enhancing cognitive outcomes.

Despite these advances, it is an undeniable fact that the derivatives of TMP developed thus far still exhibit various shortcomings and remain far from achieving excellence. It is imperative to reflect on the future direction of our efforts by analyzing the gains and losses in the development of these derivatives, particularly in enhancing neurovascular protective functions—an area where the parent compound TMP itself has demonstrated significant potential. In a recent effort, Feng et al. (2024) try to outline TMP’s pharmacokinetic challenges and describe SAR-based strategies—such as phenolic acid conjugation and prodrug design—for creating enhanced derivatives with improved efficacy and reduced toxicity. However, there is a conspicuous lack of comprehensive systematic reviews that elucidate the SAR of TMP-based derivatives specifically in the context of cerebrovascular and cognitive function. Bridging this knowledge gap is critical for guiding the next-generation of TMP-inspired drug discovery and development.

Therefore, we systematically categorize derivatives by design strategy and critically dissect the advantages and disadvantages imparted by specific structural changes on critical ADME (absorption, distribution - particularly BBB penetration, metabolism, excretion) parameters. Furthermore, we integrate discussion of advanced strategies employed to overcome these ADME limitations–such as nanodelivery systems, prodrug approaches, and targeted structural optimization–and evaluate their effectiveness. By providing this integrated analysis linking chemical structure to biological activity, ADME properties, and delivery solutions, we intend to offer actionable insights for the rational design and accelerated development of innovative TMP-based therapeutic agents targeting cerebrovascular and cognitive impairment.

## 2 Molecular structure, pharmacological profile, and SAR principles of TMP

### 2.1 The parent scaffold

**FIGURE 2:**
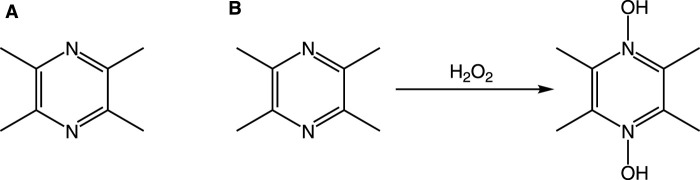
Chemical structure of TMP and its proposed interaction with H_2_O_2_. Notes: **(A)** The structure of TMP features a pyrazine ring with two nitrogen atoms at positions 1 and 4, and four methyl groups (-CH_3_) at positions 2, 3, 5, and 6. **(B)** In the presence of an oxidant like H_2_O_2_, the nitrogen atoms can be oxidized, for instance, by accepting an oxygen atom to form an N-oxide, without altering the methyl groups. This represents one of its potential antioxidant mechanisms.

The unique molecular architecture of TMP is the foundation of its diverse pharmacological activities. Its structure consists of a pyrazine core—a six-membered aromatic ring with two nitrogen atoms in para-positions—and four methyl substituents at the 2, 3, 5, and 6 positions (Figure 2).

This structure imparts a dual nature to the molecule. The pyrazine ring itself is considered the core pharmacophore, a relatively planar and electron-deficient system that underpins TMP’s broad therapeutic effects, including its vasodilatory, neuroprotective, anti-inflammatory, and antioxidant activities (Zheng et al., 2018; Jiang et al., 2020; Li et al., 2020; Ma et al., 2021; Li and Yang, 2022; Liu et al., 2022; Chen et al., 2023). Concurrently, the four symmetrically placed methyl groups increase the molecule’s lipophilicity. This enhancement is critical, as it facilitates TMP’s ability to cross the BBB and engage with a wide range of biological targets within the CNS, establishing its multi-target therapeutic potential (Wang and Wang, 2013).

However, these same structural features also introduce significant clinical limitations. The enhanced lipophilicity that aids BBB penetration also makes TMP susceptible to rapid metabolic clearance in the liver, resulting in a short biological half-life (t_1_/_2_ ≈ 2.89 h) and consequently, low bioavailability (Cai et al., 1989). This “double-edged sword” characteristic—whereby the structure enabling its therapeutic action also causes its rapid elimination—is the primary driver for the structural optimization of TMP derivatives.

### 2.2 Foundational principles of SAR-Driven optimization

The limitations of the parent TMP molecule provide a clear rationale for applying SAR principles to design superior derivatives. The goal is to retain or enhance the therapeutic efficacy of the core pyrazine pharmacophore while systematically modifying the structure to improve its pharmacokinetic profile (ADME) and potency. These research efforts are primarily guided by two integrated principles: (1) Deepening Structure-Mechanism Links: This involves understanding how a specific structural modification directly impacts a biological mechanism. For example, instead of just noting general anti-inflammatory effects, SAR analysis seeks to determine how adding a specific functional group (e.g., a phenolic acid) to the TMP scaffold creates a hybrid molecule that is a more potent inhibitor of a specific inflammatory target, such as the NF-κB signaling pathway. The goal is to translate a defined structural change into a predictable and enhanced mechanistic outcome. (2) Connecting Structure to Pharmacokinetics: This principle focuses on how structural changes directly affect the drug’s journey through the body (Wang and Wang, 2013). A key example is analyzing how replacing a methyl group with a different, bulkier moiety can create steric hindrance. This structural shield can protect the pyrazine ring from rapid metabolism by hepatic enzymes, thereby directly prolonging the molecule’s half-life and increasing its bioavailability. Here, the structural modification is rationally designed to overcome a specific pharmacokinetic barrier.

By applying these principles, researchers have developed a new generation of TMP-based compounds with enhanced activity and more favorable pharmacological profiles, providing a strong foundation for their potential clinical application in cerebrovascular and cognitive disorders.

### 2.3 Core pharmacological mechanisms of the TMP scaffold

#### 2.3.1 Linking structure to mechanism: anti-inflammatory effects

Chronic inflammation serves as a central pathogenic driver in cerebrovascular and cognitive disorders, orchestrated by pro-inflammatory cytokines including interleukin-6 (IL-6), tumor necrosis factor-α (TNF-α), and interleukin-1β (IL-1β). These mediators propagate oxidative stress, disrupt BBB integrity, and amplify neuroinflammation, culminating in neuronal damage and cognitive decline (Wang et al., 2020; Amanollahi et al., 2023). TMP demonstrates significant anti-inflammatory efficacy attributable to its unique molecular architecture, positioning it as a robust neuroprotective agent for ischemic stroke and Alzheimer’s disease (Li et al., 2018; Huang et al., 2021; Mu et al., 2023). The anti-inflammatory activity of TMP is structurally governed by its electron-deficient pyrazine ring and lipophilic methyl substituents, which collectively enable multi-pathway modulation. Specifically, the pyrazine pharmacophore directly inhibits the TLR4/NF-κB signaling cascade, suppressing nuclear translocation of transcription factors and downregulating synthesis of TNF-α, IL-1β, and IL-6, thereby attenuating inflammatory cell activation and endothelial dysfunction (Zhang et al., 2020; Jiang et al., 2022; Jiang et al., 2023; Alsfouk, 2024). Concurrently, the methyl groups at positions 2,3,5,6 enhance BBB penetration, facilitating interactions with CNS-resident microglia to promote polarization from pro-inflammatory M1 (↓IL-6, ↓CD16) to anti-inflammatory M2 phenotypes (↑Arg-1, ↑IL-10, ↑TGF-β1), effectively resolving neuroinflammation (Feng et al., 2023). The planar configuration of the pyrazine scaffold further enables crosstalk with oxidative pathways through upregulation of Sirt1/NF-κB and Nrf-2/HO-1 axes, enhancing cellular antioxidant capacity and mitigating ROS-driven inflammatory amplification (Li et al., 2019; Huo et al., 2025). Moreover, methyl group-mediated lipophilicity permits vascular compartment access, where TMP scavenges ROS and inhibits matrix metalloproteinase-9 (MMP-9), preserving tight junction proteins (occludin, claudin-5) and blocking leukocyte CNS infiltration (Tan et al., 2015). Strategic structural optimization of TMP further enhances anti-inflammatory potency, exemplified by Tetramethylpyrazine-2′-O-sodium ferulate (TSF), a hybrid derivative integrating TMP with sodium ferulate. This covalent linkage merges dual pharmacophores: the sodium ferulate moiety augments antioxidant capacity (direct ROS scavenging), while the TMP core maintains NF-κB suppression, yielding synergistic anti-inflammatory action. Critically, the polar ferulate group improves aqueous solubility and bioavailability, demonstrating concurrent pharmacokinetic/pharmacodynamic refinement (Xu et al., 2017; Zhou H. et al., 2019). Therapeutically, these structural attributes translate to neurovascular protection across disease models. In Alzheimer’s pathology, lipophilic methyl groups facilitate exosomal mRNA transfer (e.g., CDK5), while the planar pyrazine ring promotes STAT3 activation to enhance synaptic plasticity (Duan et al., 2020; Chen et al., 2024). Vascular homeostasis is restored through endothelial anti-inflammatory effects (↓IL-2/IL-6/IL-1β) (Yang et al., 2019; Ai and Zheng, 2025) and SIRT1/VEGFA-mediated reduction of ischemic infarct volume (Shu et al., 2024). Combinatorial regimens leverage TMP’s properties, such as in the Gardenia-Ligusticum Chuanxiong herb pair, where it regulates MAPK signaling and inhibits vascular apoptosis via FGFR3/AKT activation (Zhang et al., 2024b; Zhang et al., 2024a), while enhancing BMSC homing to ischemic regions through CXCR4 upregulation (Li et al., 2017). In summary, TMP’s anti-inflammatory efficacy is intrinsically governed by its pyrazine-methyI synergy, enabling concurrent targeting of cytokine networks, oxidative stress, and endothelial integrity to address core neurovascular pathology. Future development of structurally optimized derivatives and advanced delivery systems holds significant clinical promise.

#### 2.3.2 Linking structure to mechanism: antioxidant effects

Oxidative stress, driven by excessive generation of reactive oxygen species (ROS) including superoxide anions (O_2_•^-^) and hydroxyl radicals (•OH), critically contributes to neuronal injury and vascular damage (Cheung and Vousden, 2022; Teleanu et al., 2022; Angelova and Abramov, 2018; Jomova et al., 2023). Within the CNS, dysregulated enzymes like NADPH oxidase (NOX) serve as primary ROS sources, compromising brain homeostasis and vascular integrity (Zilberter et al., 2023). TMP counteracts these pathological processes through a dual antioxidant mechanism intrinsically governed by its molecular architecture. The antioxidant properties of TMP derive directly from its pyrazine scaffold, operating via complementary pathways: direct radical scavenging and indirect endogenous pathway modulation. For direct scavenging, the electron-rich pyrazine core serves as a redox-active center where hyperconjugation effects from the four methyl groups enhance nitrogen electron-donating capacity, enabling neutralization of diverse ROS including •OH and DPPH• radicals (Donkor et al., 2016). Structurally, this allows oxidation by H_2_O_2_ to form TMP-dioxide, directly consuming oxidants and preserving mitochondrial function (Li et al., 2010). Concurrently, TMP’s planar configuration facilitates indirect antioxidant effects through Nrf2/HO-1 pathway activation, promoting nuclear translocation of Nrf2 and subsequent upregulation of cytoprotective enzymes (Wang et al., 2016), while its structural compatibility with endothelial signaling upregulates DDAHII to maintain nitric oxide bioavailability and attenuate ischemia-reperfusion injury (Zhang et al., 2022; Chang et al., 2023). SAR studies demonstrate how targeted modifications enhance antioxidant efficacy, exemplified by HTMP (2-hydroxymethyl-3,5,6-trimethylpyrazine), where strategic replacement of one methyl group with a hydroxyl moiety confers dual advantages (Jiang et al., 2025). The hydroxyl group acts as a potent hydrogen donor, augmenting radical scavenging capacity, while its polarity reduces metabolic susceptibility, extending biological half-life (Chen Xin, 1996; Xuemin et al., 1998). Therapeutically, TMP’s BBB-permeable antioxidant structure mitigates neurodegenerative pathology, reducing Aβ aggregation and tau hyperphosphorylation in Alzheimer’s models via inhibition of oxidative-stress-dependent kinases like GSK-3β (Lu et al., 2017; Feng et al., 2022), while vascular protection is achieved through ROS scavenging that enhances nitric oxide bioavailability (Chang et al., 2015) and mitochondrial stabilization that reduces electron transport chain ROS leakage during ischemia-reperfusion (Ding et al., 2019; Yang et al., 2019). These findings underscore TMP derivatives as potent therapeutic candidates for oxidative stress pathologies, with future research poised to optimize clinical translation through advanced delivery systems.

#### 2.3.3 Linking structure to mechanism: regulation of programmed cell death (PCD)

Programmed cell death (PCD) pathways—including apoptosis, autophagy, and pyroptosis—play complex roles in neurodegenerative and vascular pathogenesis (Tower, 2015). While excessive apoptosis drives neuronal and endothelial loss in conditions like ischemic stroke, protective autophagy maintains cellular homeostasis by clearing damaged organelles (Sorice, 2022; Ekundayo et al., 2024). TMP and its derivatives precisely modulate these pathways through structure-dependent mechanisms (Dhaliwal et al., 2022). For apoptosis inhibition, TMP’s lipophilic methyl groups facilitate membrane penetration and mitochondrial accumulation, while its electron-rich pyrazine core scavenges ROS to prevent mitochondrial depolarization and cytochrome c release (Chang et al., 2023). Structurally, this preserves mitochondrial integrity, suppressing caspase-9/-3 activation while upregulating Bcl-2 and downregulating Bax (Cheng et al., 2009; Li et al., 2017; Tong et al., 2022). The derivative TSF exemplifies SAR-driven enhancement, where sodium ferulate conjugation improves aqueous solubility and introduces a negative surface charge that inhibits platelet aggregation, demonstrating pharmacokinetic and therapeutic dual optimization (Ji et al., 1996). Autophagy modulation is structurally augmented in derivatives like LPD, where a benzhydryl-hexahydropyrimidine side chain creates steric bulk that activates PPARγ, enhancing autophagic flux and mitochondrial energetics in Alzheimer’s models (Li et al., 2022). Pyroptosis inhibition leverages TMP’s redox-active pyrazine ring to disrupt ROS-dependent NLRP3 inflammasome activation, preventing caspase-1 cleavage and Gasdermin D pore formation that would release IL-1β/IL-18 (Zhang et al., 2022; Xu et al., 2025; Vasudevan et al., 2023). Therapeutically, these structural attributes enable multi-pathway neurovascular protection: neuronal differentiation via PI3K/Akt/Sp1 activation (Yan et al., 2015); atherosclerosis reduction through endothelial apoptosis/pyroptosis inhibition and VEGF-driven angiogenesis (Li et al., 2022; Shu et al., 2024); and derivative-enabled dual actions like T-006s concurrent apoptosis blockade and autophagy promotion in Parkinsonian models (Zhou P. et al., 2019). Thus, TMP’s molecular architecture provides a versatile scaffold for regulating cell death/survival balance in cognitive and vascular disorders.

#### 2.3.4 Linking structure to mechanism: regulation of calcium homeostasis

Calcium ion (Ca^2+^) dysregulation fundamentally drives pathophysiology in neurovascular diseases including ischemic stroke and Alzheimer’s disease (Metwally et al., 2021; Webber et al., 2023), where excessive intracellular Ca^2+^ overload triggers mitochondrial dysfunction, apoptosis, and excitotoxicity culminating in neuronal death and vascular damage (Garbincius and Elrod, 2022; Rahi and Kaundal, 2024). TMP modulates aberrant Ca^2+^ signaling through structure-dependent mechanisms anchored in two key physicochemical properties: lipophilic methyl groups facilitating membrane partitioning for direct interaction with calcium channels, and the redox-active pyrazine core protecting these channels from oxidative damage during ischemia (Hu et al., 2017). Structurally, this enables dose-dependent inhibition of pathological Ca^2+^ influx through voltage-gated channels while balancing excitatory neurotransmitter release (Li et al., 2019; Yu et al., 2019; Jin et al., 2024), thereby preserving mitochondrial function and suppressing Ca^2+^-dependent inflammatory cascades like TLR4-NLRP3 (Fu et al., 2019). SAR-driven enhancement further optimizes calcium regulation through strategic molecular hybridization, exemplified by nitro-functionalized derivatives that introduce nitric oxide (NO) donation to modulate channels via cGMP pathways, and L-carnitine conjugates that bolster mitochondrial fatty acid metabolism to intrinsically enhance Ca^2+^ buffering capacity (Wang et al., 2017). Therapeutically, these structural attributes translate to neurovascular protection through attenuated vascular smooth muscle cell (VSMC) hypercontraction and potently inhibited platelet aggregation (Li and Yang, 2022), establishing calcium homeostasis regulation as a cornerstone of TMP’s therapeutic efficacy against cognitive and vascular pathologies.

#### 2.3.5 Linking structure to mechanism: protection of BBB integrity

The BBB constitutes a highly selective endothelial border critical for cerebral homeostasis, whose breakdown through tight junction (TJ) disruption is a pathological hallmark of neurological disorders from ischemic stroke to Alzheimer’s disease (Gu et al., 2011). TMP exhibits dual structure-enabled functions: its small molecular dimensions and methyl group-conferred lipophilicity facilitate efficient BBB penetration, while the redox-active pyrazine core counteracts barrier-degrading processes through antioxidant and anti-inflammatory actions. Mechanistically, TMP preserves TJ proteins (occludin, claudin-5) by inhibiting matrix metalloproteinase (MMP)-mediated extracellular matrix degradation (Gong et al., 2019), while simultaneously suppressing inflammatory signaling cascades (e.g., JAK/STAT pathway) and cytokine-driven MMP upregulation that increase barrier permeability (Xu et al., 2017; Jin et al., 2021). Structural optimization is exemplified by TSF (tetramethylpyrazine-2′-O-sodium ferulate), where the TMP scaffold delivers conjugated ferulate antioxidant moieties across compromised barriers during ischemia-reperfusion, yielding enhanced local ROS scavenging, reduced BBB disruption, diminished infarct volume, and improved neurological outcomes (Xu et al., 2017). This targeted delivery paradigm demonstrates how rational molecular hybridization leverages TMP’s innate biodistribution properties to amplify therapeutic efficacy at pathological sites, reinforcing its multifunctional potential in neurovascular disorders where BBB integrity is compromised.

## 3 SAR-driven design of novel TMP derivatives for neurovascular protection

The inherent therapeutic potential of the TMP scaffold, combined with its significant pharmacokinetic limitations, has driven extensive research into the rational design of novel derivatives. The primary goal of these medicinal chemistry efforts is to retain or enhance the core pharmacophore’s beneficial activities while systematically overcoming its drawbacks, such as poor bioavailability and short half-life. This chapter reviews several key classes of TMP derivatives, organized by their distinct design strategies, to illustrate how SAR principles have been applied to create next-generation compounds with superior efficacy for cerebrovascular and cognitive disorders.

### 3.1 Strategy 1: Molecular hybridization for multi-target efficacy (T-006)

#### 3.1.1 Design rationale

The design of T-006 is a prime example of a molecular hybridization strategy. The goal was to create a single chemical entity with multi-target capabilities by combining the structural features of two distinct pharmacophores: the neuroprotective scaffold of J147 and the vasoprotective TMP moiety. The specific aim was to replace a biologically inactive portion of the J147 molecule with TMP, thereby creating a novel compound with synergistic and enhanced neuroprotective and cognitive-enhancing effects.

#### 3.1.2 Structural modification and SAR

**FIGURE 3:**
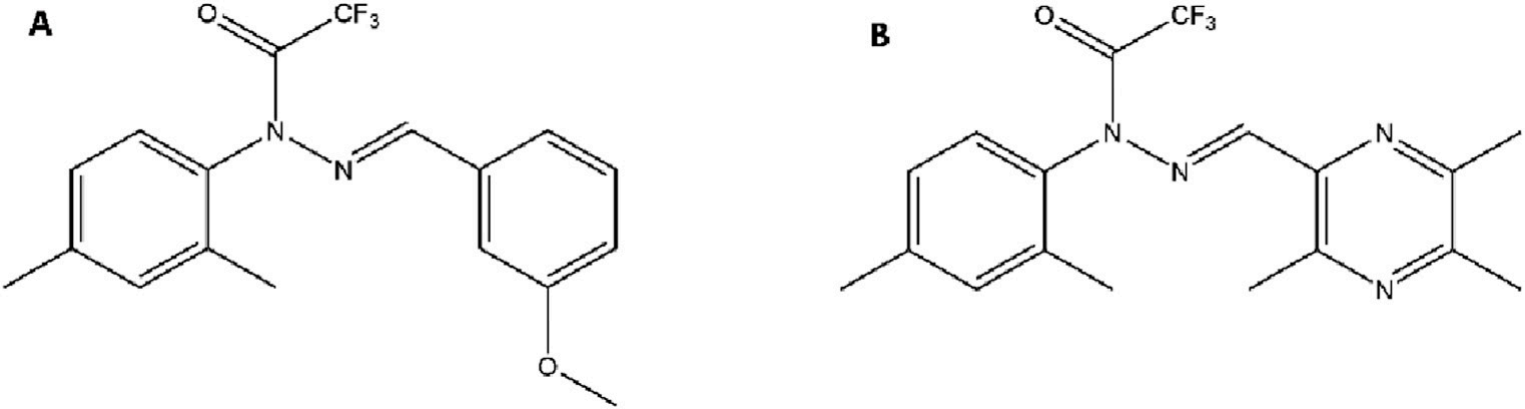
Chemical Structures of the Parent Compound J147 and the Hybrid Derivative T-006. Notes: **(A)** J147 features a central pyrazole ring linked to a bis(4-methoxyphenyl) group on one side and a phenyl ring with a trifluoroacetamide group on the other. **(B)** In T-006, one of the methoxyphenyl groups of J147 is strategically replaced with a TMP moiety, connected via a C-C single bond to the pyrazole core.

T-006 was synthesized by covalently linking TMP to the core structure of J147. Specifically, the methoxyphenyl group on J147, which was found to be non-essential for its activity, was replaced with a TMP molecule via a stable C-C bond (Figure 3). This strategic modification resulted in a hybrid compound with a significantly different physicochemical profile. The introduction of polar functional groups within the core scaffold optimizes the lipid-water partition coefficient, enhancing its ability to penetrate the BBB while improving its overall bioavailability compared to the parent TMP (Wu et al., 2019; Jiehong et al., 2021).

#### 3.1.3 Pharmacological superiority and mechanisms

This structure-guided hybridization translates into markedly superior pharmacological activity. T-006 exhibits significantly enhanced neuroprotection (EC_50_ = 59.4 nM) compared to its parent compounds (Xu et al., 2016). This potency is quantified primarily by determining the half-maximal effective concentration (EC_50_) or half-maximal inhibitory concentration (IC_50_). EC_50_measures the concentration of an agonist required to elicit 50% of the maximal response, whereas IC_50_ measures the concentration of an inhibitor needed to suppress 50% of a biological process (Li et al., 2015). When the EC_50_/IC_50_ values of a derivative drop by several-fold or more, the compound is considered a promising candidate for further development (Zhang et al., 1999). Its multi-target mechanism includes: (1) Enhanced Mitochondrial Function: T-006 activates the PKA/Akt/GSK-3β and CREB/PGC-1α signaling networks, promoting mitochondrial biogenesis and restoring cellular energy balance (Zhou H. et al., 2019). (2) Potent Antioxidant and Anti-inflammatory Effects: It powerfully regulates both the mTOR and Nrf2/HO-1 pathways, providing more robust protection against oxidative stress and neuroinflammation than TMP alone (Zhang G. et al., 2024). (3) Synaptic Repair and Neuroregeneration: T-006 upregulates key synaptic proteins like BDNF and synaptophysin, promoting synaptic plasticity and long-term cognitive recovery in models of Alzheimer’s and Parkinson’s disease (Chen et al., 2020).

Despite its advantages, the increased lipophilicity of T-006 compared to TMP raises concerns about potential hepatic accumulation and long-term hepatotoxicity (Qi et al., 2016). Addressing this challenge requires further SAR-driven refinement. A logical next step would be to introduce controlled polarity into the T-006 scaffold—for example, by adding small, hydrophilic groups (e.g., hydroxyl or carboxyl moieties) at non-critical positions. Such modifications could reduce overall lipophilicity and mitigate liver accumulation without compromising BBB permeability or therapeutic efficacy. This represents a classic medicinal chemistry approach to “fine-tuning” a lead compound for a better safety profile.

### 3.2 Strategy 2: Incorporating a bioactive moiety for enhanced radical scavenging (TBN and TN-2)

#### 3.2.1 Design rationale

This strategy focuses on incorporating a well-known bioactive functional group—a nitrone—directly onto the TMP scaffold. Nitrones are potent “spin traps,” capable of scavenging highly reactive and damaging free radicals. However, standalone nitrone drugs have historically been limited by poor BBB penetration. The design rationale for Tetramethylpyrazine Nitrone (TBN) was to create a hybrid molecule that uses the BBB-permeable TMP scaffold as a “delivery vehicle” to transport the highly effective nitrone trap into the CNS (Cancela et al., 2020; Zhou et al., 2023).

#### 3.2.2 Structural modification and SAR

**FIGURE 4:**
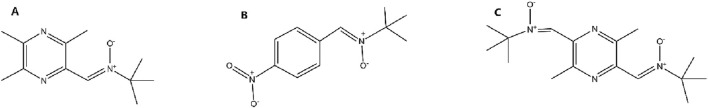
Chemical Structures of a Nitrone Spin Trap, TBN, and its Dual-Nitrone Derivative TN-2. Notes: **(A)** In TBN, the methyl group at position 3 of the TMP ring is oxidized to form a nitrone functional group. **(B)** A generic nitrone structure, which acts as a free radical “spin trap.” **(C)** In TN-2, the methyl groups at both the 3 and 6 positions of the TMP ring are converted into nitrone functional groups, creating a dual-trap molecule.

TBN is created by modifying one of the methyl groups on the TMP pyrazine ring to form a nitrone functional group (Figure 4A). This modification masterfully combines the properties (Figure 4B) of both parent structures. The resulting hybrid molecule, TBN, retains the lipophilicity and small size necessary to readily cross the BBB, achieving therapeutic concentrations in brain tissue that are approximately 68% of plasma levels—a significant improvement over traditional nitrones (Zhang et al., 2016). For TMP derivatives targeting the central nervous system (CNS), adequate blood–BBB permeability is essential. Assessment typically involves the following approaches: *in silico* models based on physicochemical properties (e.g., logP, molecular weight, polar surface area) can predict BBB penetration (Morales et al., 2017).

**FIGURE 5:**
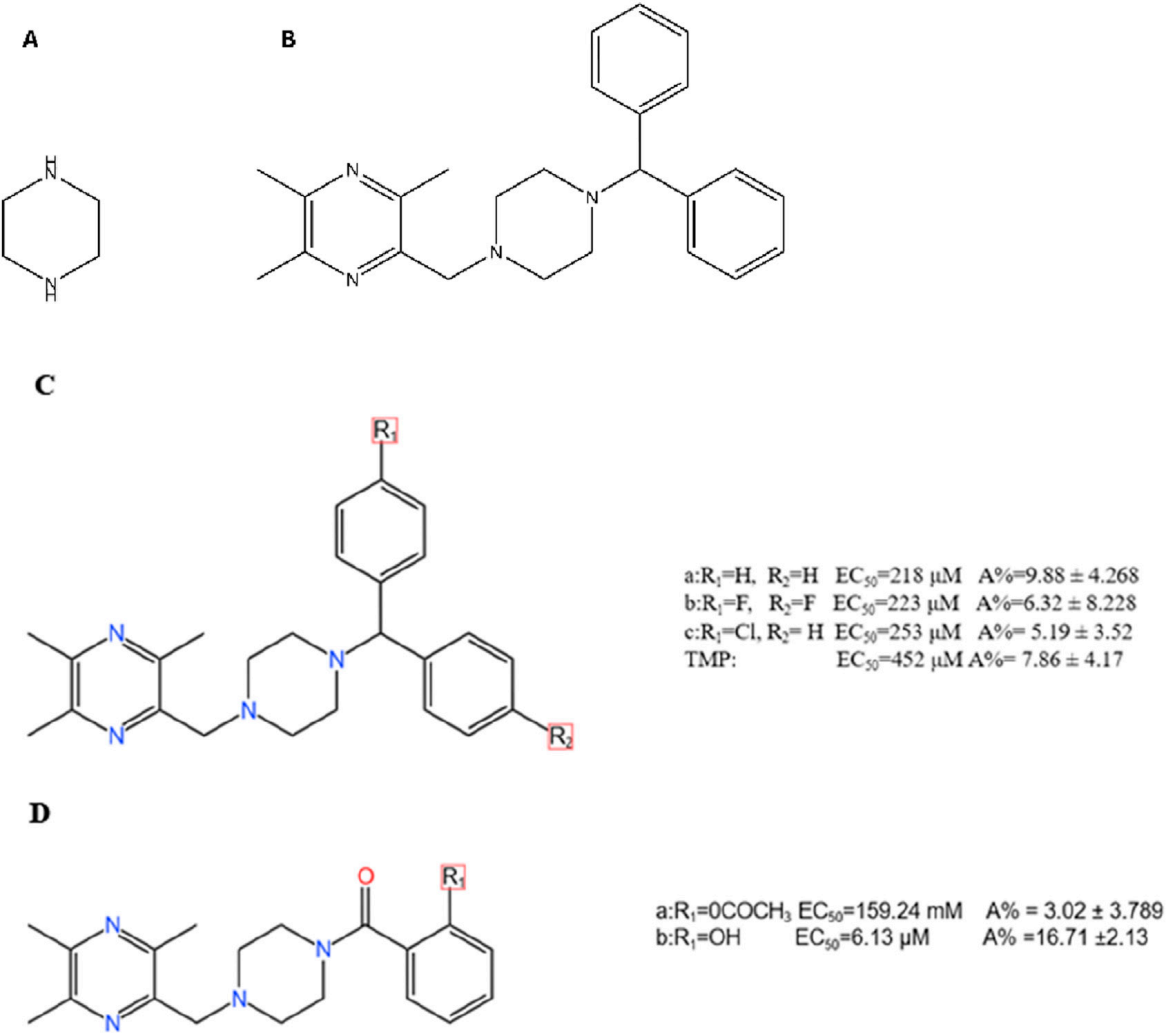
Chemical Structures of the Piperazine Scaffold and Representative TMP-Piperazine Derivatives. Notes: **(A)** The piperazine scaffold, a six-membered ring with two modifiable nitrogen atoms. **(B)** A TMP-piperazine derivative where the piperazine ring links the TMP moiety to a biphenylmethyl group. **(C,D)** Other examples of TMP-piperazine derivatives where various substituents are attached to the piperazine ring to modulate biological activity.

Building on this success, a second-generation derivative, TN-2, was developed (Figure 5C). The SAR logic here was straightforward: if one nitrone trap is effective, two may be even better. TN-2 incorporates two nitrone groups at positions 3 and 6 of the pyrazine ring (Figure 4C). This modification was designed to increase the molecule’s stoichiometric capacity for scavenging cytotoxic free radicals like hydroxyl radicals, superoxide, and peroxynitrite (Xu et al., 2014).

#### 3.2.3 Pharmacological superiority and mechanisms

The incorporation of the nitrone moiety endows TBN with powerful, multi-faceted neuroprotective effects that surpass those of TMP alone: (1) Potent ROS Scavenging: The nitrogen-oxygen bond of the nitrone is highly effective at neutralizing ROS, directly reducing oxidative stress-induced neuronal damage (Sun et al., 2008). (2) Comprehensive Neuroprotection: TBN modulates Ca^2+^ influx, balances the Bax/Bcl-2 ratio to prevent apoptosis, and activates key pro-survival signaling pathways, including Nrf2/HO-1 and PI3K/Akt (Zhang et al., 2016; Zhang et al., 2018). (3) Enhanced Autophagy and Aβ Clearance: In AD models, TBN activates the AMPK/mTOR/ULK1 autophagy pathway, which is critical for clearing toxic protein aggregates like Aβ (Zhou et al., 2024). (4) Mitochondrial Protection: TBN modulates the AMPK/mTORC1 signaling axis to optimize mitochondrial biogenesis and improve cellular energy metabolism, contributing to its anti-aging effects (Nie et al., 2024; Zhang T. et al., 2024).

#### 3.2.4 Clinical potential and future directions

TBN represents an innovative and successful molecular design. Its excellent BBB penetration, potent multi-target neuroprotective actions, and favorable safety and tolerability profile in early clinical studies make it a highly promising candidate for acute ischemic stroke and chronic neurodegenerative diseases (Cucchiara et al., 2009; Zhu et al., 2024). The development of dual-nitrone derivatives like TN-2 further demonstrates the potential for SAR-guided optimization to enhance radical-scavenging potency. Continued clinical validation is essential to fully realize the therapeutic potential of this class of compounds.

### 3.3 Strategy 3: Using a linker scaffold for pharmacokinetic and pharmacodynamic optimization (piperazine derivatives)

#### 3.3.1 Design rationale

This strategy employs piperazine, a versatile six-membered heterocyclic ring, as a central linker or scaffold. The rationale is to use piperazine’s unique properties to systematically optimize the parent TMP molecule. The two nitrogen atoms of the piperazine ring offer distinct, modifiable sites: one can anchor the TMP moiety, while the other provides a handle to attach various other pharmacophores (Figure 5A). This design allows medicinal chemists to fine-tune key properties like solubility, basicity, and receptor affinity, thereby improving both the pharmacokinetic profile and the biological activity of the resulting derivatives (Clark et al., 2014; Jain et al., 2021; Brito et al., 2019). The goal is to create novel molecules where the piperazine linker bridges TMP with another functional group to achieve synergistic or enhanced therapeutic effects.

#### 3.3.2 Structural modification and SAR

Early work revealed that the pharmacological effects of TMP were dependent on the pyrazine core, while its pharmacokinetics and toxicity were linked to its substituents (Guohui et al., 1996). This insight spurred the development of piperazine-linked derivatives (Figure 5C). (1) Introducing Benzoyl Groups for Antiplatelet Activity: In one series of compounds, various substituted benzoyl groups were attached to the piperazine linker (Figure 5D). SAR studies showed that this modification dramatically enhanced antiplatelet activity. Compound 5D(b), for example, emerged as a highly potent inhibitor (EC_50_ = 6.13 μM), far exceeding the efficacy of TMP itself. This demonstrates that using the piperazine linker to introduce an aromatic benzoyl moiety creates a new pharmacophore with strong anti-aggregating properties (Cheng et al., 2007). (2) Introducing Biphenylmethyl Groups for PPARγ Agonism: In a different approach, a bulky biphenylmethyl group was attached to the piperazine linker (Figure 5B). This structural design was intended to create a molecule capable of interacting with the nuclear receptor PPARγ, a key regulator of glucose metabolism with known neuroprotective roles (Prashantha Kumar et al., 2020). This strategy was highly successful, yielding novel PPARγ agonists that are not present in the parent TMP molecule (Li S. et al., 2022).

#### 3.3.3 Pharmacological superiority and mechanisms

The use of the piperazine linker has successfully generated derivatives with novel and enhanced mechanisms of action: (1) Enhanced Antiplatelet and Vasoprotective Effects: The benzoyl-piperazine derivatives achieve their potent antiplatelet effects by effectively inhibiting pathways involved in platelet aggregation, demonstrating a clear gain of function compared to TMP (Cheng et al., 2007). Other derivatives have shown strong cytoprotective effects against H_2_O_2_-induced endothelial cell damage (Cheng et al., 2009). (2) Novel PPARγ Agonism for Neuroprotection: The biphenylmethyl-piperazine derivatives act as novel PPARγ agonists. By activating this pathway, they improve mitochondrial autophagy and glucose metabolism in the hippocampus, leading to the alleviation of cognitive deficits in AD models (Li Z. et al., 2022). This represents the successful introduction of an entirely new mechanism of action through rational drug design.

#### 3.3.4 Limitations and future directions

While versatile, the piperazine scaffold has known liabilities. Its inherent basicity can lead to lysosomotropism (accumulation in lysosomes), which can alter intracellular drug distribution and potentially cause off-target effects. Furthermore, achieving an optimal balance between BBB permeability, target engagement, and a long plasma half-life remains a significant challenge. Future research must focus on fine-tuning the physicochemical properties of these derivatives—perhaps by modifying the piperazine ring itself or by exploring bioisosteric replacements—to mitigate these risks while retaining the impressive therapeutic gains.

### 3.4 Strategy 4: Hybridization with phenolic acids for synergistic antioxidant and anti-inflammatory effects

#### 3.4.1 Design rationale

This strategy involves creating hybrid molecules by linking the TMP scaffold to various naturally occurring phenolic acids, such as vanillic acid, ferulic acid, or salicylic acid. These phenolic acids are known to possess intrinsic neuroprotective, antioxidant, and anti-inflammatory properties (Krzysztoforska et al., 2019; Siddiqui et al., 2019; Ahmad Bainmahfouz et al., 2021). The design rationale is to create a single, synergistic molecule that combines the BBB-penetrating and vasoprotective effects of TMP with the potent antioxidant and anti-inflammatory activities of a phenolic acid. The goal is to produce derivatives with enhanced, multi-faceted neuroprotective efficacy and improved pharmacokinetic profiles compared to administering either compound alone.

#### 3.4.2 Structural modification and SAR

**FIGURE 6:**
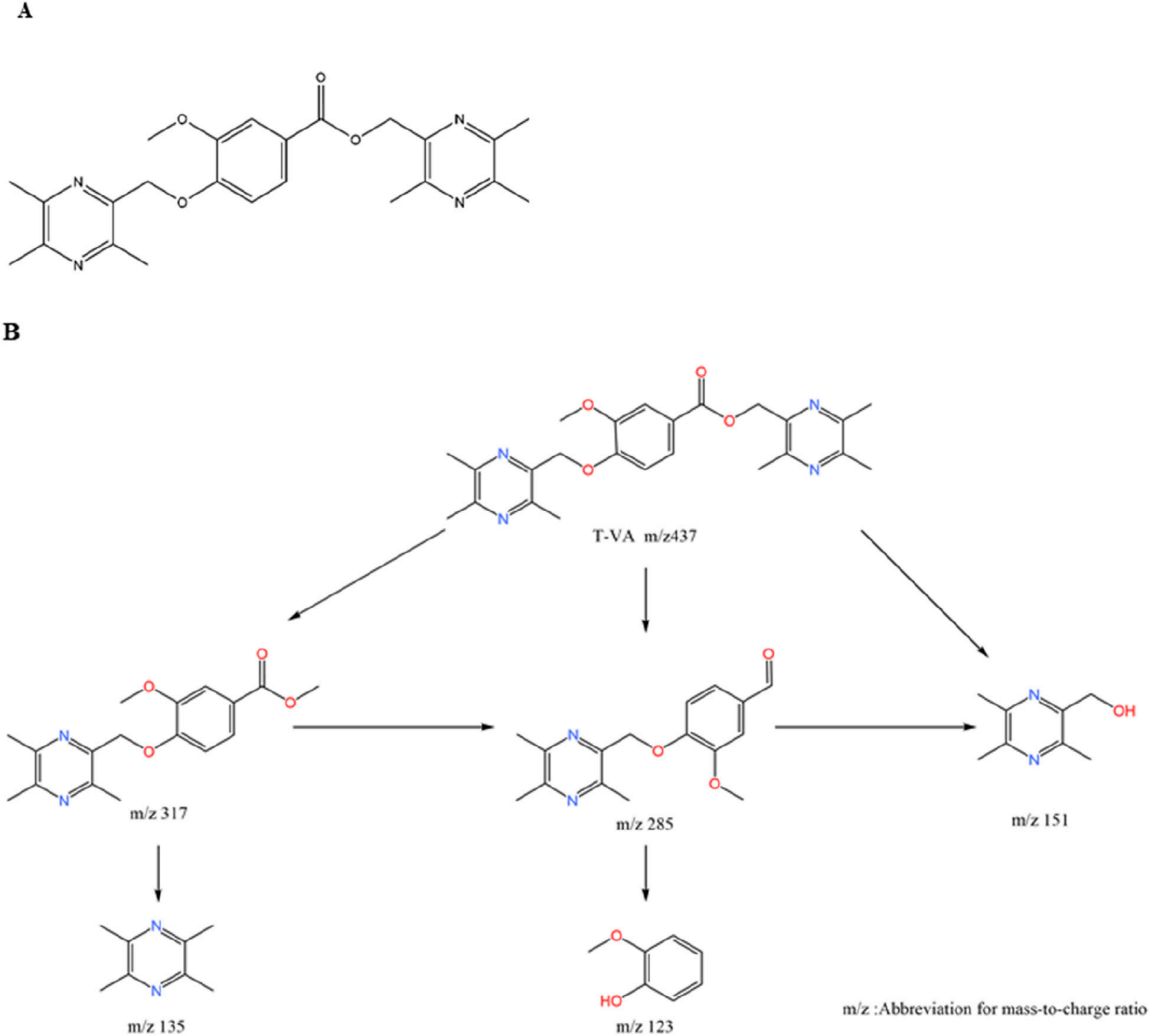
Chemical Structure of T-VA and its Major Metabolite. Notes: **(A)** T-VA is a dimeric structure where a central vanillic acid molecule is linked via its carboxyl group to two separate TMP-piperazine units. **(B)** The major metabolite of T-VA is formed by the metabolic cleavage of one of the TMP-piperazine units, leaving a single unit attached to the vanillic acid core.

These hybrids are typically formed by connecting TMP to the phenolic acid via an ester, ether, or amide linkage. Linker and Substituent Position: SAR studies have revealed critical structural rules for maximizing efficacy. First, ether-linked derivatives consistently demonstrate superior neuroprotective effects and metabolic stability compared to their ester-linked counterparts. Second, the position of the substituent on the phenolic acid’s benzene ring is crucial; para-substituted compounds exhibit stronger neuroprotective activity than those with ortho- or meta-substitutions (Wang et al., 2013). Example 1: T-VA: The derivative T-VA is a dimeric compound where two TMP-piperazine units are linked to a central vanillic acid molecule (Figure 6A). It exhibits exceptionally strong protective effects on damaged neuronal cells (EC_50_ = 4.249 μM), significantly outperforming TMP (Zhang et al., 2017). Example 2: TSF: The derivative TSF (tetramethylpyrazine-2′-O-sodium ferulate) links TMP with ferulic acid. This modification not only combines antioxidant effects but also alters the molecule’s electrostatic properties, which significantly inhibits platelet aggregation—a key benefit for treating ischemic conditions (Zhou P. et al., 2019; Wang et al., 2023).

#### 3.4.3 Pharmacological superiority and mechanisms

The phenolic acid hybrids demonstrate enhanced and synergistic mechanisms of action: (1) Enhanced Neuroprotection via Anti-inflammation: T-VA and related derivatives exert their potent neuroprotective effects by downregulating key inflammatory pathways, including the expression of NF-κB and cyclooxygenase-2 (COX-2) (Wang et al., 2013). Similarly, TSF has been shown to inhibit the TLR4/NF-κB signaling pathway, reducing neuroinflammation and brain edema in stroke models (Zhou H. et al., 2019). (2) Metabolite Activity (Prodrug Effect): T-VA itself is active, but it also functions as a prodrug. *In vivo*, it is metabolized to a major metabolite that retains significant neuroprotective activity (Chenze et al., 2017) (Figure 6B). This extends the therapeutic duration of action, as the active metabolite continues to provide protection after the parent drug is cleared (Zhang et al., 2017). (3) Synergistic Vasoprotection: Hybrids like T-VA have been shown to upregulate VEGF expression and Ca^2+^-Mg^2+^ ATPase activity, promoting angiogenesis and restoring ionic balance, while TSF provides a direct and potent antiplatelet effect (Li et al., 2015).

While highly effective, the metabolic stability of the linker—particularly the ester linkage—remains a key challenge for some of these derivatives. The observation that ether-linked compounds are more stable provides a clear direction for future optimization. Future research should focus on designing phenolic acid hybrids with more robust linkers or exploring advanced prodrug strategies to ensure controlled release of the active moieties *in vivo*. Fine-tuning the balance between hydrophilicity (for solubility) and lipophilicity (for BBB penetration) will also be critical to maximizing the clinical potential of this promising class of compounds.

## 4 Advanced drug delivery systems to overcome pharmacokinetic hurdles

While the rational design of derivatives described in Chapter 3 can significantly enhance the therapeutic efficacy of the TMP scaffold, many of these new chemical entities still face pharmacokinetic challenges. Issues such as short half-life, poor aqueous solubility, or potential off-target toxicities (e.g., the risk of hepatic accumulation with highly lipophilic compounds like T-006) can limit their clinical translation. To overcome these hurdles, researchers are developing advanced drug delivery systems designed to control the release, improve the stability, and precisely target the delivery of TMP and its derivatives, thereby maximizing their therapeutic potential while minimizing systemic side effects.

The SAR-driven design of novel TMP derivatives for neurovascular protection systematically optimizes the core scaffold through four key strategies: (1) Molecular hybridization (e.g., T-006 fuses TMP with J147 via C-C bond, replacing J147s non-essential methoxyphenyl to enhance neuroprotection,but requiring polarity adjustments to mitigate hepatotoxicity); (2) Bioactive moiety incorporation (e.g., TBN/TN-2 add nitrone groups at pyrazine C3/C6, boosting ROS scavenging and BBB penetration); (3) Linker scaffold engineering (e.g., piperazine-linked derivatives attach benzoyl/biphenylmethyl groups to enable antiplatelet activity or PPARγ agonism); (4) Phenolic acid hybridization (e.g., T-VA uses ether-linked vanillic acid for synergistic anti-inflammation, with para-substitution critical for efficacy. Advanced delivery systems (ROS-responsive nanoparticles, conductive hydrogels, focused ultrasound) address residual PK challenges (e.g., hepatic accumulation, short half-life) by enabling targeted CNS release and sustained local action, collectively overcoming TMP’s limitations while amplifying its neurovascular benefits (Table 1).

**TABLE 1:** Impact of key structural modifications on TMP derivatives’ pharmacokinetics (PK) and pharmacodynamics (PD).

Modification type	Representative functional groups	Primary PK impact	Primary PD impact	Supporting rationale
Increased Polarity	-OH (e.g., HTMP), -COOH	↑ Aqueous Solubility ↑ Plasma Half-life (t_1_/_2_), Altered Metabolism	↑ Direct Radical Scavenging (if -OH), Potentially Altered Target Profile	Reduced LogP; -OH acts as H-donor; Altered metabolic susceptibility (e.g., slower glucuronidation)
Bulky Lipophilic	Aryl (e.g., phenyl), Complex alkyl	Variable Solubility ↑ Metabolic Stability (t_1_/_2_) ↓ BBB Penetration	Potential for ↑ Target Affinity/Selectivity, New Mechanisms (e.g., PPARγ activation by LPD)	Steric hindrance protects core; New interactions with hydrophobic binding pockets
Polar/Ionizable	Piperazine, Amino groups	↑ Solubility (especially salt forms) ↑ Plasma Half-life (t_1_/_2_) Altered Distribution	Enhanced interactions with charged targets (e.g., enzymes, receptors), New mechanisms	Introduces H-bond acceptors/donors and basic centers; Improved PK profile Potential for ionic interactions
Electron-Withdrawing	Nitro (-NO_2_), Nitrone (C=N^+^(O^−^)R)	Variable (Nitro: ↓Solubility; Nitrone: ↑Reactivity)	↑ Antioxidant Capacity (Radical Trapping), Potential NO Release (Nitro groups as prodrugs)	Enhanced radical scavenging (nitrone as spin trap) Metabolic conversion of nitro to NO.
Bioactive Hybrid	Ferulic acid (e.g., TSF), Carnitine	Variable Solubility (depends on conjugate) ↑ Molecular Weight	Synergistic Effects: ↑Antioxidant ↑Anti-inflammatory, New Mechanisms (e.g., Platelet inhibition in TSF)	Combines TMP’s core actions with conjugate’s actions (e.g., ferulate’s antioxidant; carnitine’s mitochondrial support).
Prodrug Linker	Ester, Amide	↑ Oral Absorption, ↑ Solubility, Masked Activity	Activity dependent on cleavage to release active moiety	Improves formulation and absorption; Protects active molecule until site-specific release

### 4.1 Nanoparticle-based targeted delivery

Nano-drug delivery systems (NDDS) utilize carriers on the nanometer scale to encapsulate therapeutic agents. This strategy offers several key advantages for TMP-based compounds: it can protect the drug from premature degradation, improve the solubility of poorly soluble derivatives, and, most importantly, achieve targeted delivery to specific tissues. Mechanism and Application:

In a state-of-the-art example, researchers developed nanoparticles with a shell made of a polymer containing reactive oxygen species (ROS)-responsive linkers to encapsulate TMP. The nanoparticle surface was then decorated with a peptide (CFLFLF) that specifically binds to formyl peptide receptors (FPR) on neutrophils. In the inflammatory environment of ischemia-reperfusion injury, these nanoparticles are carried by neutrophils directly to the site of damage. The high local concentration of ROS then breaks the nanoparticle shell, releasing the TMP payload precisely where it is needed. This “smart” delivery system dramatically improves drug accumulation at the target site (Mu et al., 2023). Addressing Derivative Limitations: This approach is particularly promising for potent but potentially toxic derivatives like T-006. By encapsulating the lipophilic drug, nanoparticles can prevent its accumulation in the liver and ensure it is released primarily in the brain, significantly improving its safety profile.

### 4.2 Conductive hydrogels for sustained local release

For site-specific injuries, such as ischemic stroke or spinal cord injury, maintaining a high local concentration of a therapeutic agent over an extended period is crucial for promoting tissue repair. Conductive hydrogels have emerged as ideal carriers for this purpose. Mechanism and Application: These hydrogels have a three-dimensional network structure and biomechanical properties similar to natural neural tissue (Xu et al., 2020). A gelatin methacrylate-polypyrrole-TMP (GMPT) conductive hydrogel has been developed that can be implanted at the site of injury. The hydrogel maintains the structural integrity of TMP while providing slow, sustained release over time. In spinal cord injury models, this system demonstrated significant efficacy in promoting neural function recovery (Deng et al., 2024). Addressing Derivative Limitations: This technology is perfectly suited for delivering derivatives designed for neuroregeneration. By providing a continuous, localized supply of the drug, it can support long-term processes like neurite outgrowth and synaptic repair without requiring frequent systemic administration.

### 4.3 Focused ultrasound for on-demand, precise delivery

Focused ultrasound (FUS) offers unparalleled spatiotemporal control over drug delivery. By non-invasively focusing ultrasound waves on a specific region of the brain, it can transiently and safely open the BB or create microchannels in cell membranes (sonoporation). Mechanism and Application: When combined with systemically administered microbubbles, FUS can temporarily increase the permeability of the BBB, allowing drugs like TMP or its derivatives to enter the brain in much higher concentrations, but only at the targeted location. Studies have shown that this ultrasound-mediated delivery enhances TMP’s protective effects against oxidative stress (Zhang et al., 2014; Zhang et al., 2018). Addressing Derivative Limitations: This on-demand, reversible delivery method is a powerful tool for maximizing efficacy while minimizing systemic exposure. It allows clinicians to deliver a potent drug precisely when and where it is needed, overcoming the general challenge of getting drugs into the CNS and reducing the risk of side effects associated with high systemic doses.

## 5 Translational challenges and future directions

TMP and its derivatives exhibit significant neuroprotective effects and have shown promising efficacy in multiple models of neurodegenerative diseases, including PD and AD (Feng et al., 2024). However, despite their favorable *in vitro* and animal-model performance, clinical translation still faces numerous challenges, chief among them metabolic instability, hepatotoxicity, and a lack of readiness for human trials (Liu et al., 2025). Moreover, critical issues such as off-target toxicity, interspecies pharmacokinetic differences, and formulation challenges have not been fully addressed, potentially hindering future clinical application.

Metabolic instability represents a major barrier to clinical translation. Many compounds that demonstrate robust efficacy in animal models fail in humans due to rapid or aberrant metabolism leading to loss of activity or toxic metabolites. For example, T-006 may be metabolized *in vivo* by cytochrome P450 enzymes, whose activity can vary markedly between species, potentially resulting in altered clearance rates and safety profiles in humans compared to preclinical models (Guengerich, 2006). Indeed, the short *in vivo* half-life of TBN limits its sustained action *in vivo* (Zhao et al., 2016). Although definitive evidence of T-006–induced hepatotoxicity is lacking, its reliance on hepatic cytochrome P450 metabolism underscores the need to investigate its metabolic fate and potential impact on liver function.

Off-target toxicity is another critical concern in drug development. Prior failures of anticancer agents in clinical trials often stemmed from unintended interactions with proteins other than the intended target, leading to unacceptable toxicity (Lin et al., 2019). While TMP derivatives display strong neuroprotective activity, it remains to be determined whether they provoke toxic effects in non-neural tissues. In particular, T-006s activation of PI3K/AKT/mTOR and Nrf2 pathways—key regulators of cell survival, metabolism, and stress responses—could perturb physiological processes in other organs, increasing the risk of adverse events (Zhang G. et al., 2024).

Interspecies pharmacokinetic differences further complicate the transition from animal models to human subjects. Variations in absorption, distribution, metabolism, and excretion between species can lead to discrepancies in bioavailability and clearance. For instance, although T-006 may exhibit adequate bioavailability and half-life in rodent models, accelerated metabolism in humans could result in rapid clearance and reduced therapeutic efficacy. Such differences may also be influenced by the formulation type—whether oral, controlled-release, or other delivery systems—which affects absorption kinetics and tissue distribution (Bae et al., 2005).

Formulation challenges are likewise pivotal. Researchers have explored several advanced delivery strategies—such as porous osmotic pumps, microemulsions, and transdermal patches—to enhance TMP derivative stability and bioavailability, yet clinical evidence remains sparse (Mei et al., 2008; Wei et al., 2012; Tang et al., 2013). Poor aqueous solubility of T-006, for example, may necessitate nanotechnology-based formulations or liposomal encapsulation to improve systemic exposure. The chosen formulation can profoundly affect both pharmacokinetics and safety, underscoring the importance of appropriate drug-delivery design for successful clinical outcomes.

Despite the compelling neuroprotective data in preclinical systems, the clinical-trial readiness of TMP derivatives warrants further evaluation. Optimal trial design—including dose selection, safety monitoring, and patient stratification—relies on comprehensive preclinical data. Factors such as age, sex, and genetic background can influence pharmacokinetics and pharmacodynamics in human populations and must be considered in trial protocols. Ultimately, rigorous investigation of metabolic pathways, off-target profiles, pharmacokinetic behavior, and formulation strategies, followed by well-controlled clinical studies, is essential to establish the safety and efficacy of TMP and its derivatives. Only by addressing these challenges can these compounds realize their potential as effective neuroprotective agents, offering new hope for the treatment of cognitive and neurovascular disorders.

## 6 Summary and prospects

This review has chronicled the remarkable evolution of TMP from a single active monomer into a versatile and highly adaptable pharmacophore for treating complex neurovascular and cognitive disorders. While the parent TMP molecule possesses intrinsic multi-target protective effects—including anti-inflammatory, antioxidant, and calcium-regulating activities—its clinical utility is hampered by poor pharmacokinetics. The core theme of this review is the power of medicinal chemistry and SAR principles to systematically overcome these limitations.

Through strategic molecular design, researchers have developed next-generation derivatives with vastly superior properties. Key successful strategies include: (1) Molecular Hybridization: Creating multi-target agents like T-006, which combines distinct pharmacophores to achieve synergistic efficacy. (2) Bioactive Moiety Incorporation: Developing potent radical scavengers like TBN, which leverages the TMP scaffold as a brain-penetrant delivery vehicle for a nitrone “trap.” (3) Synergistic Conjugation: Designing hybrids like TSF (TMP-ferulic acid), which couple TMP with phenolic acids to enhance both vasoprotective and antioxidant effects. Furthermore, the development of advanced drug delivery systems—such as targeted nanoparticles and localized hydrogels—provides a parallel and complementary approach to enhance the therapeutic index of these compounds, ensuring they reach their target site in effective concentrations while minimizing systemic exposure.

The journey of TMP is far from over. To translate the impressive preclinical success of its derivatives into clinical reality, future research should focus on several critical and innovative directions: (1) Next-Generation Medicinal Chemistry: The field should move beyond simple conjugation towards more sophisticated chemical biology approaches. This includes designing covalent or allosteric modulators of specific enzymes or receptors to achieve greater target selectivity and potency. Exploring novel modalities like proteolysis-targeting chimeras (PROTACs) that use the TMP scaffold to recruit E3 ligases for the targeted degradation of pathogenic proteins (e.g., misfolded tau) represents a truly next-generation strategy. (2) Exploring Novel Biological Targets: While current derivatives effectively manage downstream effects like oxidative stress and inflammation, future work should aim to modulate more fundamental upstream drivers of neurodegeneration. Promising targets include the glymphatic system (the brain’s waste clearance pathway) and the accumulation of senescent cells within the neurovascular unit. Designing TMP derivatives that can enhance glymphatic clearance or selectively eliminate senescent cells could offer disease-modifying, rather than merely symptomatic, benefits. (3) Overcoming Hurdles in Clinical Translation: The path to clinical approval requires addressing several key challenges. A top priority is the identification and validation of predictive biomarkers that can identify patient populations most likely to respond to these multi-target agents. Clinical trial designs must also evolve, perhaps employing genetic stratification or biomarker-based enrichment strategies. Finally, for complex derivatives or drug-device combinations (e.g., TSF, GMPT hydrogels), ensuring the scalability and cost-effectiveness of manufacturing will be paramount for successful commercialization and patient access.

By pursuing these advanced strategies, the scientific community can build upon the solid foundation of TMP chemistry to develop truly transformative therapies for Alzheimer’s disease, stroke, and other devastating neurovascular disorders.
